# Long non-coding RNA BLACAT1 promotes breast cancer cell proliferation and metastasis by miR-150-5p/CCR2

**DOI:** 10.1186/s13578-019-0274-2

**Published:** 2019-01-30

**Authors:** Xiaopeng Hu, Yun Liu, Yaying Du, Teng Cheng, Wenfei Xia

**Affiliations:** 1 Department of Breast and Thyroid Surgery, Division of General Surgery, Tongji Hospital, Tongji Medical College, Huazhong University of Science and Technology, 1095 Jiefang Avenue, Wuhan, 430030 Hubei China; 2 Department of ENT, Tongji Hospital, Tongji Medical College, Huazhong University of Science and Technology, Wuhan, Hubei China

**Keywords:** BLACAT1, Breast cancer, miR-150-5p, CCR2

## Abstract

**Background:**

Breast cancer was dangerous to women health. A growing number of evidences indicate that long non-coding RNAs (lncRNAs) have many functions in the development and progression of breast cancer and may serve as the markers of diagnosis or prognosis. BLACAT1 is one of lncRNA and the roles of it in breast cancer is not clear. In this study, it is aimed to explore the roles and molecular mechanisms of BLACAT1 in breast cancer.

**Results:**

We found BLACAT1 took part in breast cancer cell aggressive phenotype. The real-time PCR result showed that BLACAT1 was up-regulated in tumor tissues compared to adjacent normal tissues. The molecular mechanism experiments demonstrated that BLACAT1 down-regulation suppressed the proliferation and metastasis of human breast cancer cells by regulating miR-150-5p targeting CCR2. The clinical studies indicated that lack of BLACAT1 was related to tumor size, metastasis. Conclusion: The present study verified the involvement of the BLACAT1 in the mediation of cell survival and metastasis through miR-150-5p targeting CCR2 in breast cancer cells.

## Introduction

Breast cancer, the most common malignant disease for women, is dangerous to women healthy. Although the modern diagnostic and therapeutic methods are developed, the mortality and morbidity of breast cancer remains a higher level [1]. In the pathophysiological progression of breast cancer, there are many molecules such as growth factors, cytokines, non-coding RNAs and others involve in the various biological progresses [2, 3]. Understanding of the molecular mechanisms of breast cancer is benefit for finding new targets of breast cancer therapy. So, it is urgent for the researchers to understand the regulatory network in breast cancer.

Long non-coding RNAs (LncRNAs) are a large heterogeneous class of transcripts longer than 200 nucleotides with limited protein-coding potential ability [4]. The studies showed that lncRNAs have many functions in various pathophysiological processes [5, 6]. The dysregulation of lncRNAs plays pivotal roles in many kinds of diseases, particularly in cancer [7, 8]. The lncRNAs circulating in serum/plasma are relatively stable because they are not degraded by RNase even in the complicated environment in vivo [9]. Thus, the lncRNAs may be acted as potential diagnostic or prognostic markers in multiple types of cancer [9]. BLACAT1, locates on human chromosome 1q32.1 and has a transcript of 2616 kb with just one exon [10]. BLACAT1 was reported in cervical cancer, gastric cancer, papillary thyroid carcinoma, colorectal cancer [11–14]. However, the regulatory and molecular mechanism of BLACAT1 in breast cancer are still unclear.

In this study, we evaluated the regulatory roles of BLACAT1 in breast cancer. The research verified the involvement of BLACAT1 in accelerating cell survival and metastasis through miR-150-5p targeting CCR2 in breast cancer cells.

## Materials and methods

### Clinical tissue samples

From March 2015 to August 2016, patients with breast cancer received surgical treatment in the Tongji Hospital, Tongji Medical College, Huazhong University of Science and Technology (Wuhan, China). The tumor tissues were rapidly frozen at − 80 °C for study. This study was approved by the Research Ethics Committee of the Tongji Hospital. The informed written consents were collected from all patients.

### Quantitative real-time polymerase chain reaction (qRT-PCR)

Total RNA was extracted from cells or tissue according to specification (Promega, Madison, WI., USA). The RNA concentrations were measured at 260/280 nm using ultraviolet spectrophotometer by the standard of OD260/OD280 ratio of 1.6**–**2.0. cDNA was reversely transcribed from RNA using SuperScript First**-**Stand Synthesis system (Invitrogen, Carlsbad, Calif, US). qRT**-**PCR was performed using ABI7500 quantitative PCR instrument. Primer sequences were synthesized by Sangon Biotech (Shanghai) as following: BLACAT1 forward, 5′**-**CAAGAGGAGCCGGCTTAGCATCTA**-**3′, reverse 5′**-**ACGGTTCCAGTCCTCAGTCAG**-**3′; miR**-**150-5p, forward, 5′**-**ACACTCCAGCTGGGCTGGTACAGGCCTGGGG**-**3′, reverse, 5′**-**GGGCATACATCGGCTAATACA**-**3′; CCR2, forward: 5′**-**TATCAGAAATACCAACGAGAGC**-**3′ reverse 5′**-**TTGGTCCACTAGTGTGAACAG**-**3′; GAPDH, forward: 5′**-**TCCACCACCCTGTTGCTGTA**-**3′, reverse: 5′**-**ACCACAGTCCATGCCATCAC**-**3′. Finally, the RNA expression levels were quantified with SYBR (Applied Biosystems, USA) on 7500 Real**-**Time PCR System (Applied Biosystems, USA). The relative expression was normalized to the expression of GAPDH using 2^−∆∆*C*t^ method. The experiments were performed in triplicate.

### Plasmid construction and transfection

Full**-**length complementary DNA of CCR2 was synthesized by GenScript Biomedical (Shanghai, China) and cloned into the pcDNA3.1 (+) vector (Invitrogen) according to the manufacturer’s instructions. A total of 5 × 10^5^ cells were seeded into each well of a 6**-**well plate and transfected with plasmids upon reaching 80%**–**90% confluence. Recombinant plasmid pcDNA**-**CCR2 and negative control plasmid (pcDNA**-**NC) were constituted and, respectively, transfected into cells using Lipofectamine 2000 (Invitrogen) according to the instruction manual. Transfection of BLACAT1 siRNA, miR**-**150-5p or the controls was performed using Lipofectamine 2000.

### Cell proliferation assay

Breast cancer cells were transfected with BLACAT1 siRNA, miR-150-5p or the control with or without CCR2 plasmids and then 10 μl Cell count kit-8 solution (CCK-8, Dojindo, Japan) was added to each well at the indicated time. Then, the cells were incubated at 37 °C for 2 h. The absorbance of the cells in every well at 490 nm was measured using a spectrophotometer.

### Colony formation assay

Breast cancer cells were transfected with siRNAs or plasmids. The cells were seeded into six well plates with high DMEM completed growth medium. After 14 days in culture, cells were fixed with 90% carbinol for 30 min and stained with 0.1% crystal violet (Beyotime, Shanghai, China). Then, cells were washed with PBS and taken photos. Colonies containing more than 50 cells were manually counted.

### Scratch wound assay

The cells with plasmids or siRNAs transfection or the controls with 2 × 10^5^ in every well were plated into a 12-well plate and incubated to reach confluence. The monolayer was scratched using a tip, washed with serum-free medium to remove detached cells and photographed at 24 h later.

### Invasion assay

The inserts in the transwell system were coated with 50 μl Matrigel (BD Biosciences, Franklin Lakes, NJ, USA). Cells (5 × 10^4^) were suspended in 100 μl serum-free medium and then seeded on the upper floor of Transwell chambers (Corning, USA). 500 μl serum with 20% FBS were added into the lower chamber. The cells were incubated at 37 °C with 5% CO_2_ for 2 days. The un-invaded cells were wiped with a cotton swab, and invaded cells were fixed in methanol and stained with 0.1% crystal violet. The number was counted under a microscope.

### Western blot

The total protein was lysed by RIPA buffer (Sigma**-**Aldrich) added with protease inhibitors cocktail (Roche). After that, the isolated protein was transferred to sodium dodecyl sulphate**–**polyacrylamide gel electrophoresis (SDS**-**PAGE), and then to PVDF membrane (Millipore, Billerica, MA, USA). The membranes were washed with TBST and blocked with 5% non**-**fat milk powder and incubated with primary antibody, anti**-**ABCB1 (1:1000 dilution, Abcam), at room temperature for 2 h or overnight at 4 °C. The membrane was incubated with second antibody (horseradish peroxidase**-**conjugated goat anti**-**rabbit) at room temperature for 2 h. Finally, these blots were detected using an EZ**-**ECL chemiluminescence detection kit for HRP (Biological Industries, Beit**-**Haemek, Israel).

### Luciferase reporter assay

SKBR3 cells (1 × 10^4^) were seeded into each well of 48**-**well plate and co-transfected with luciferase reporter (10 ng) and miR**-**150-5p mimics (50 nmol/l) or negative control using Lipofectamine 2000 (Invitrogen). 48 h after transfection, luciferase activity assay was measured using the Dual**-**Luciferase Reporter Assay System (Promega, Madison, WI, USA) and GloMax 20/20 LUMINOMETER (promega). Renilla luciferase activity was normalized against firefly luciferase activities. Results represented the triplicate independent experiments.

### Statistical analysis

All data were generated from tripartite independent experiments and presented as the mean ± SD. Statistical analyses were performed using SPSS 18.0 and GraphPad software. Differences between groups were analyzed using Student’s *t* test or Chi square test analysis. Statistical significance was set as *P* < 0.05.

## Results

### Long non-coding RNA BLACAT1 expression in breast cancer tissues

**Fig. 1:**
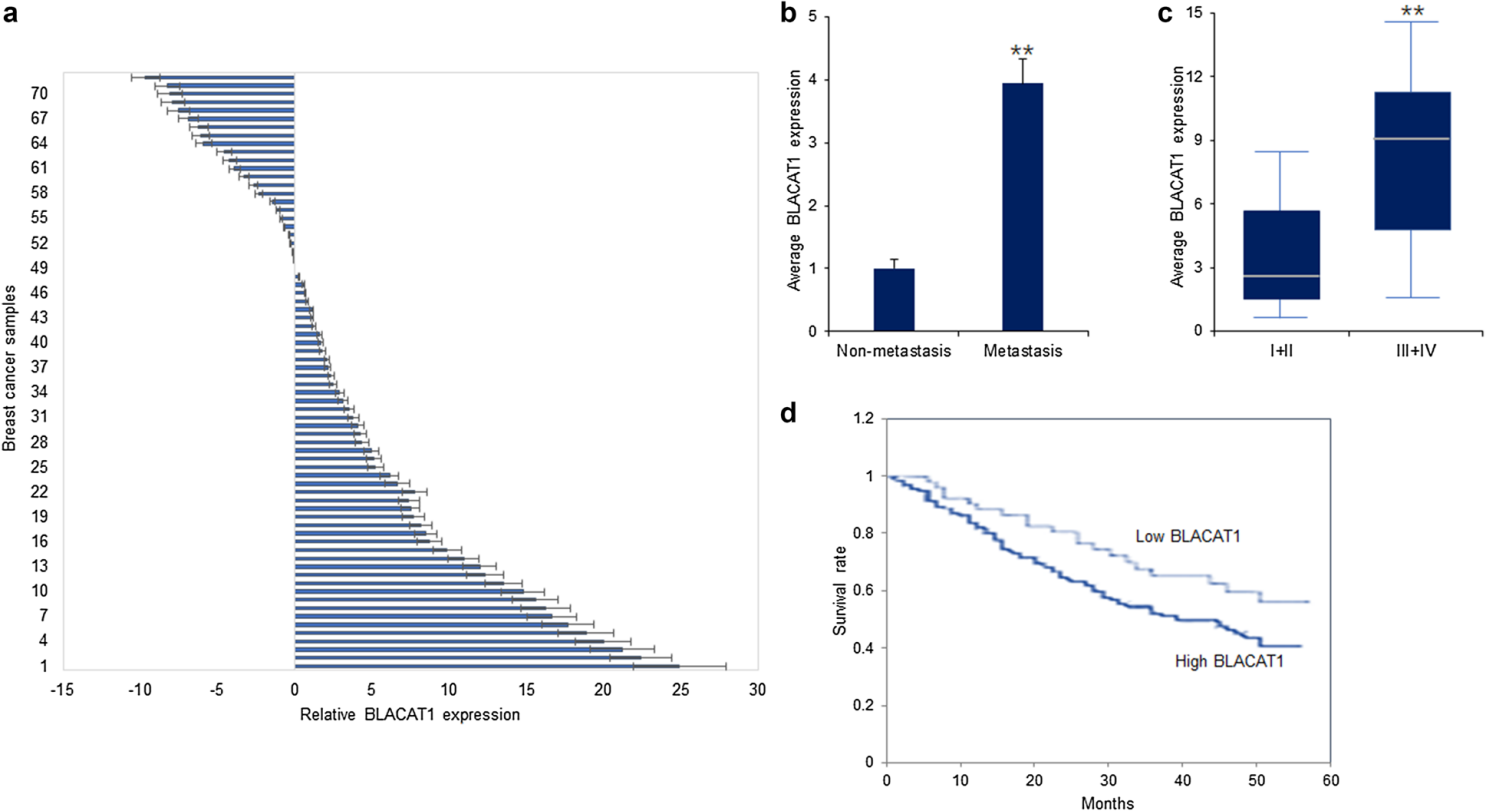
Long non-coding RNA BLACAT1 expression in breast cancer tissues. **a** BLACAT1 levels were higher in breast cancer tissues. 72 breast cancer tissues were prepared for RNA extraction. BLACAT1 expression was measured by real time RT-PCR. BLACAT1 expression in breast cancer tissues was compared to BLACAT1 expression in their compared normal tissues. **b** BLACAT1 expression was associated with breast cancer metastasis. The data was analyzed from BLACAT1 expression in metastatic tissues (n = 42) and non-metastatic tissues (n = 30). **c** BLACAT1 expression was associated with breast cancer tumor stage. The data was analyzed from BLACAT1 expression in different stage of tumor. **d** BLACAT1 expression was associated with the survival time of breast cancer patients. When the expression of BLACAT1 was more than twofold when comparing with its expression in adjacent normal tissues. Based on the statistical calculation, the fold changes of BLACAT1 expression in breast cancer tissues comparing to the expression of it in normal tissues more than twofold meant high BLACAT1 expression; less than 0.5-folds meant low BLACAT1 expression

To investigate the roles of BLACAT1 in breast cancer, BLACAT1 expression in breast cancer tissues was measured by real time RT-PCR. It was shown that BLACAT1 levels were higher in breast cancer tissues than their compared normal tissues (Fig. 1a). Further clinical analysis showed that higher BLACAT1 expression in breast cancer tissues was associated with metastasis (Fig. 1b and Table 1) and tumor staging (Fig. 1c and Table 1). The survival time was shorter in breast cancer patients with high BLACAT1 expression (Fig. 1d). The data suggested that BLACAT1 maybe function as a tumor promoting long non-coding RNA in breast cancer.

**Table 1:** The relationship between BLACAT1 expression and the clinic characters of breast cancer

Parameters	LncRNA BLACAT1		P
	Low (%)	High (%)	
Age (years)			
> 60	17	15	0.865
< 60	18	22	
Tumor size (cm)			
> 2	24	17	0.727
< 2	16	15	
ER			
Positive	14	19	0.345
Negative	19	20	
PR			
Positive	24	16	0.052*
Negative	18	14	
HER2			
Positive	16	14	0.085
Negative	23	19	
TNM staging			
I + II	12	23	0.013*
III + IV	15	22	
Lymph node metastasis			
Positive	15	24	0.027*
Negative	12	21	

*ER* estrogen receptor, *PR* progesterone receptor

* Significantly difference

### BLACAT1 suppressed miR-150-5p expression in breast cancer cells

**Fig. 2:**
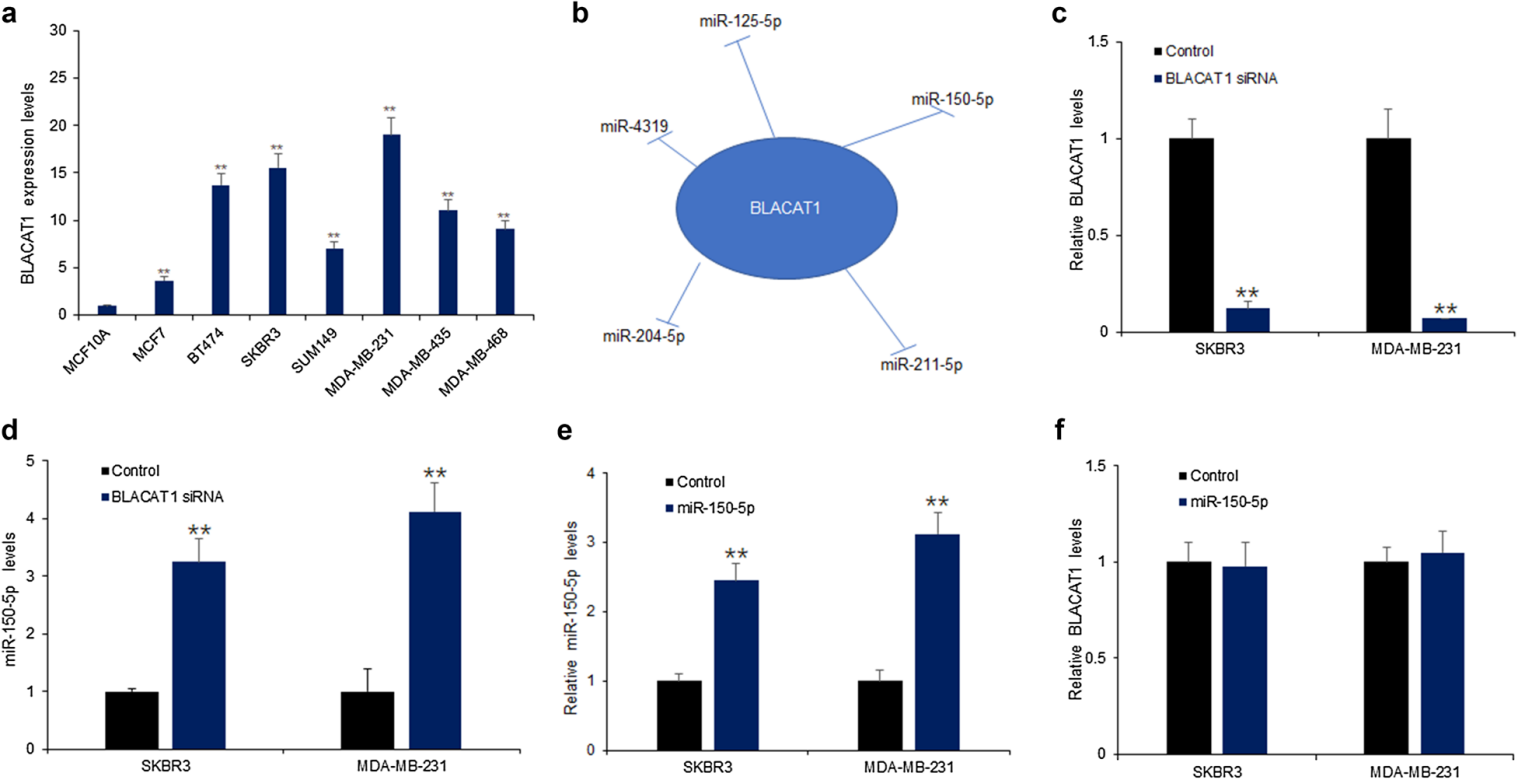
BLACAT1 suppressed miR-150-5p expression in breast cancer cells. **a** BLACAT1 expression in breast cancer cell lines. Total RNA was isolated from breast cancer cells and performed for BLACAT1 expression analysis by real time RT-PCR. **b** The prediction of miRNAs associated with BLACAT1. **c** BLACAT1 expression was effectively down-regulated in SKBR3 and MDA-MBA-231 cells with BLACAT1 siRNA transfection. **d** miR-150-5p was up-regulated in SKBR3 and MDA-MB-231 cells with BLACAT1 down-regulation. **e** miR-150-5p expression was effectively up-regulated in SKBR3 and MDA-MBA-231 cells with miR-150-5p transfection. **f** MiR-150-5p showed no influence on the expression level of BLACAT1 in SKBR3 and MDA-MB-231 cells

For exploring the regulatory roles of BLACAT1 in breast cancer cells, firstly, BLACAT1 expression was measured in MCF10A cells and seven breast cancer cell lines including MCF-7, BT474, SKBR3, SUM149, MDA-MB-231, MDA-MB-435 and MDA-MB-468. The data demonstrated that BLACAT1 level in MCF10A cells was the lowest and its levels in SKBR3 and MDA-MB-231 cells were the highest (Fig. 2a). To know the potential miRNAs which were regulated by BLACAT1, the database predicted that BLACAT1 might regulate miR-125-5p, miR-4319, miR-211-5p, miR-204-5p, miR-150-5p expression (Fig. 2b). As shown in Fig. 2c, miR-150-5p was up-regulated in SKBR3 and MDA-MB-231 cells with BLACAT1 down-regulation. But, there was no influence of miR-150-5p on BLACAT1 expression in the above cell lines (Fig. 2d). The results indicated that miR-150-5p might be a sponge of BLACAT1 in breast cancer cells.

### BLACAT1 promoted breast cancer cell survival and metastasis via miR-150-5p

**Fig. 3:**
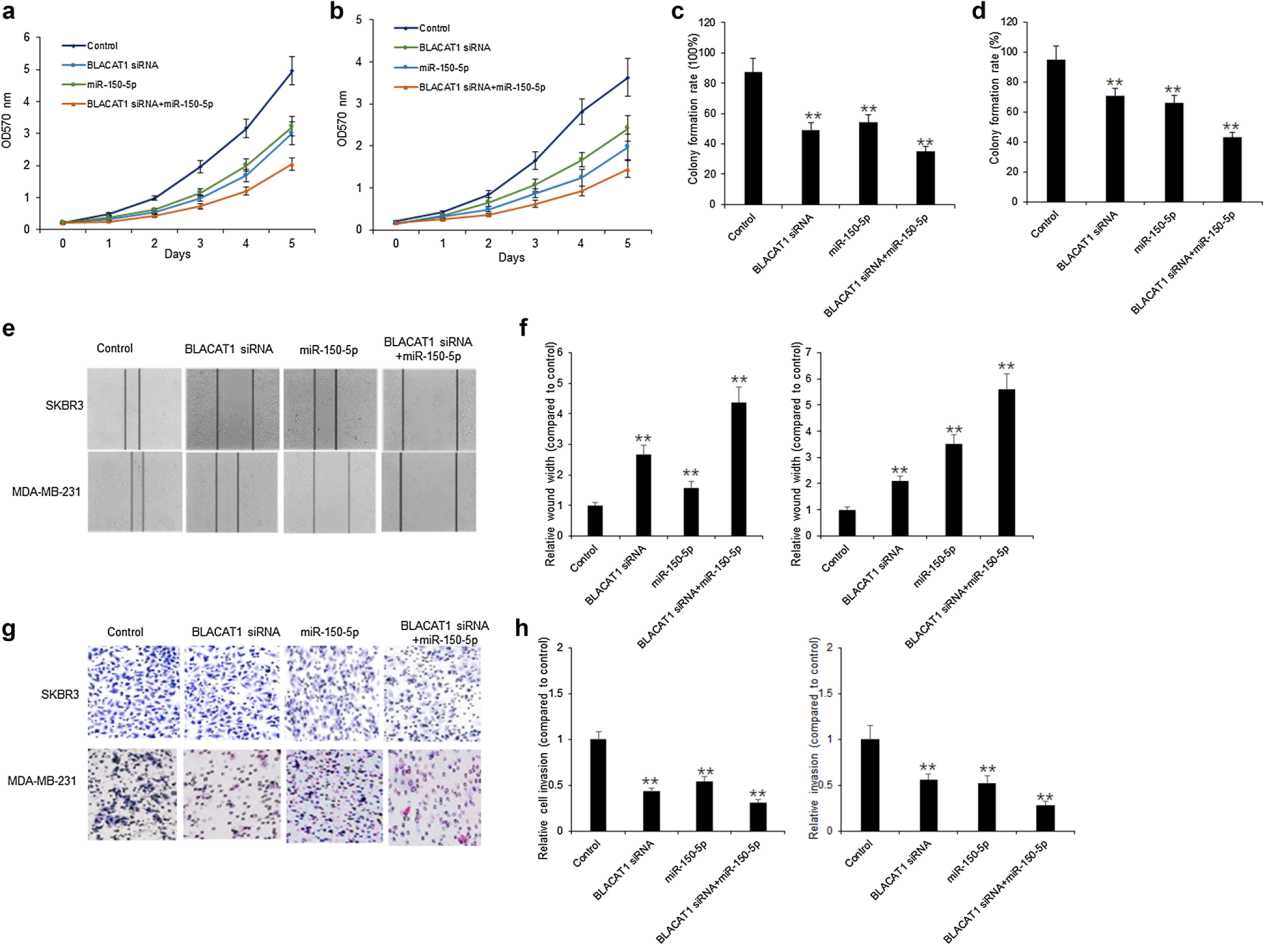
BLACAT1 promoted breast cancer cell survival via miR-150-5p. **a**, **b** MTT assay showed that cell proliferation was dramatically inhibited by knockdown of BLACAT1 or up-regulation of miR-150-5p in SKBR3 and MDA-MB-231 cells. **c**, **d** SKBR3 and MDA-MB-231 cell survival abilities were assayed by colony formation. SKBR3 and MDA-MB-231 cells were transfected with BLACAT1 siRNA or miR-150-5p mimics for 24 h, seeded in the 6-well plates culturing for 2 weeks and colonies were counted. **e**, **f** SKBR3 and MDA-MB-231 cell migration was assayed by wound-healing assay. SKBR3 and MDA-MB-231 cells were transfected with BLACAT1 siRNA or miR-150-5p mimics for 24 h and cell migration was analyzed. **g**, **h** SKBR3 and MDA-MB-231 cell migration was assayed by invasion assay. SKBR3 and MDA-MB-231 cells were transfected with BLACAT1 siRNA or miR-150-5p mimics for 24 h and cell invasion was analyzed

To assess the cellular survival of BLACAT1 in breast cancer cells, SKBR3 and MDA-MB-231 cells were transfected with BLACAT1 siRNAs or miR-150-5p. MTT assay was used to assess cell survival of SKBR3 and MDA-MB-231 cells with BLACAT1 siRNAs or miR-150-5p. The data showed that down-regulation of BLACAT1 decreased cell survival rates in SKBR3 and MDA-MB-231 cells with miR-150-5p overexpression (Fig. 3a, b). The data from colony formation assay showed that down-regulation of BLACAT1 reduced cell colonies of SKBR3 and MDA-MB-231 cells with or without miR-150-5p overexpression (Fig. 3c, d). The data indicated that BLACAT1 down-regulation suppressed breast cancer cell growth by sponging miR-150-5p.

To assess the cellular metastasis ability of BLACAT1 in breast cancer cells, SKBR3 and MDA-MB-231 cells were transfected with BLACAT1 siRNAs or miR-150-5p. Wound healing assay was used to assess cell survival of SKBR3 and MDA-MB-231 cells with BLACAT1 siRNAs or miR-150-5p. The data showed that down-regulation of BLACAT1 decreased cell migration in SKBR3 and MDA-MB-231 cells with miR-150-5p overexpression (Fig. 3e, f). Transwell assay showed that down-regulation of BLACAT1 decreased cell migration in SKBR3 and MDA-MB-231 cells with miR-150-5p overexpression (Fig. 3g, h). The data indicated that BLACAT1 down-regulation suppressed breast cancer cell metastasis by sponging miR-150-5p.

### miR-150-5p suppressed CCR2 expression in breast cancer cells

**Fig. 4:**
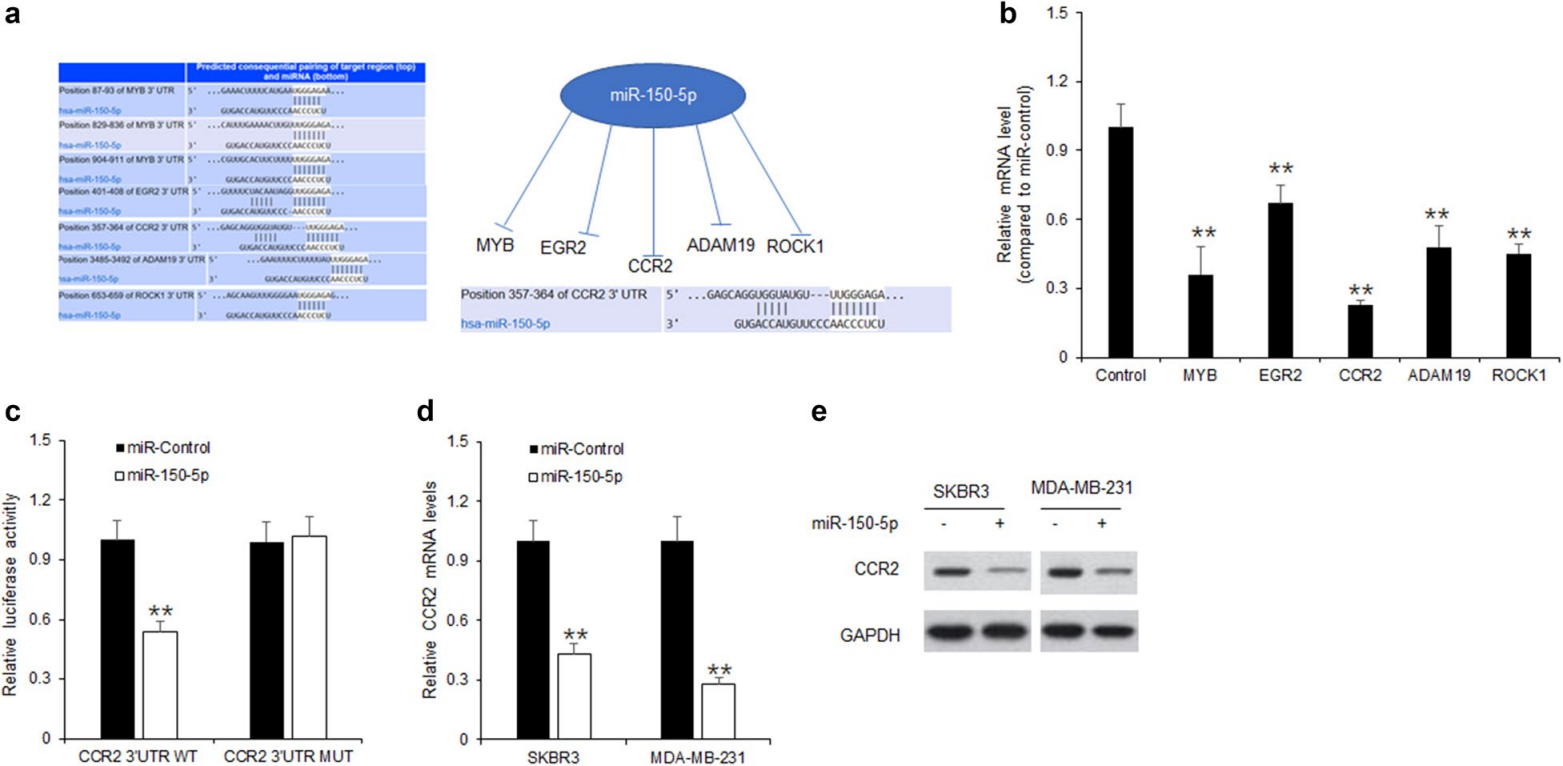
miR-150-5p suppressed CCR2 expression in breast cancer cells. **a** The predicted target genes of miR-150-5p. **b** The mRNA levels in SKBR3 cells with miR-150-5p transfection. SKBR3 cells were transfected with miR-150-5p and total RNA was extracted for qRT-PCR analysis. **c** MiR-150-5p significantly inhibited luciferase activity of wild type reporter for CCR2, The putative sequences of miR-150-5p and CCR2 with a binding site. **d** MiR-150-5p down-regulated CCR2 mRNA levels in SKBR3 and MDA-MB-231 cells. SKBR3 and MDA-MB-231 cells were transfected with miR-150-5p mimics and total RNA was extracted for real time RT-PCR analysis. **e** MiR-150-5p down-regulated CCR2 protein levels in SKBR3 and MDA-MB-231 cells by western blotting. SKBR3 and MDA-MB-231 cells were transfected with miR-150-5p mimics and total protein was extracted for western blot analysis

To explore the mechanism of BLACAT1 in regulating breast cancer cell proliferation and metastasis by down-regulating miR-150-5p, the target genes of miR-150-5p were predicted (LncRNA Disease) and five of them were selected for further verification. As shown in Fig. 4a, the top five target genes were listed. To confirm the most closely related to miR-150-5p, SKBR3 cells were transfected with miR-150-5p mimics and five predicted target genes including MYB, EGR2, CCR2, ADAM19 and ROCK1 by Targetscan were confirmed using qRT-PCR. The result indicated that CCR2 mRNA decreased more than the other genes including MYB, EGR2, ADAM19 and ROCK1 in SKBR3 cells with miR-150-5p up-regulation (Fig. 4b). Using luciferase assay, CCR2 was further confirmed as a target gene of miR-150-5p in SKBR3 cells, miR-150-5p inhibited the luciferase activity of reporter vector containing the wide-typed binding sites of CCR2 in SKBR3 cells, but not the mutated CCR2 3′-UTR (Fig. 4c). MiR-150-5p down-regulated CCR2 mRNA levels in SKBR3 and MDA-MB-231 cells by qRT-PCR (Fig. 4d). MiR-150-5p down-regulated CCR2 protein levels in SKBR3 and MDA-MB-231 cells by western blotting (Fig. 4e).

### BLACAT1 promoted breast cancer cell proliferation and metastasis by miR-150-5p/CCR2

**Fig. 5:**
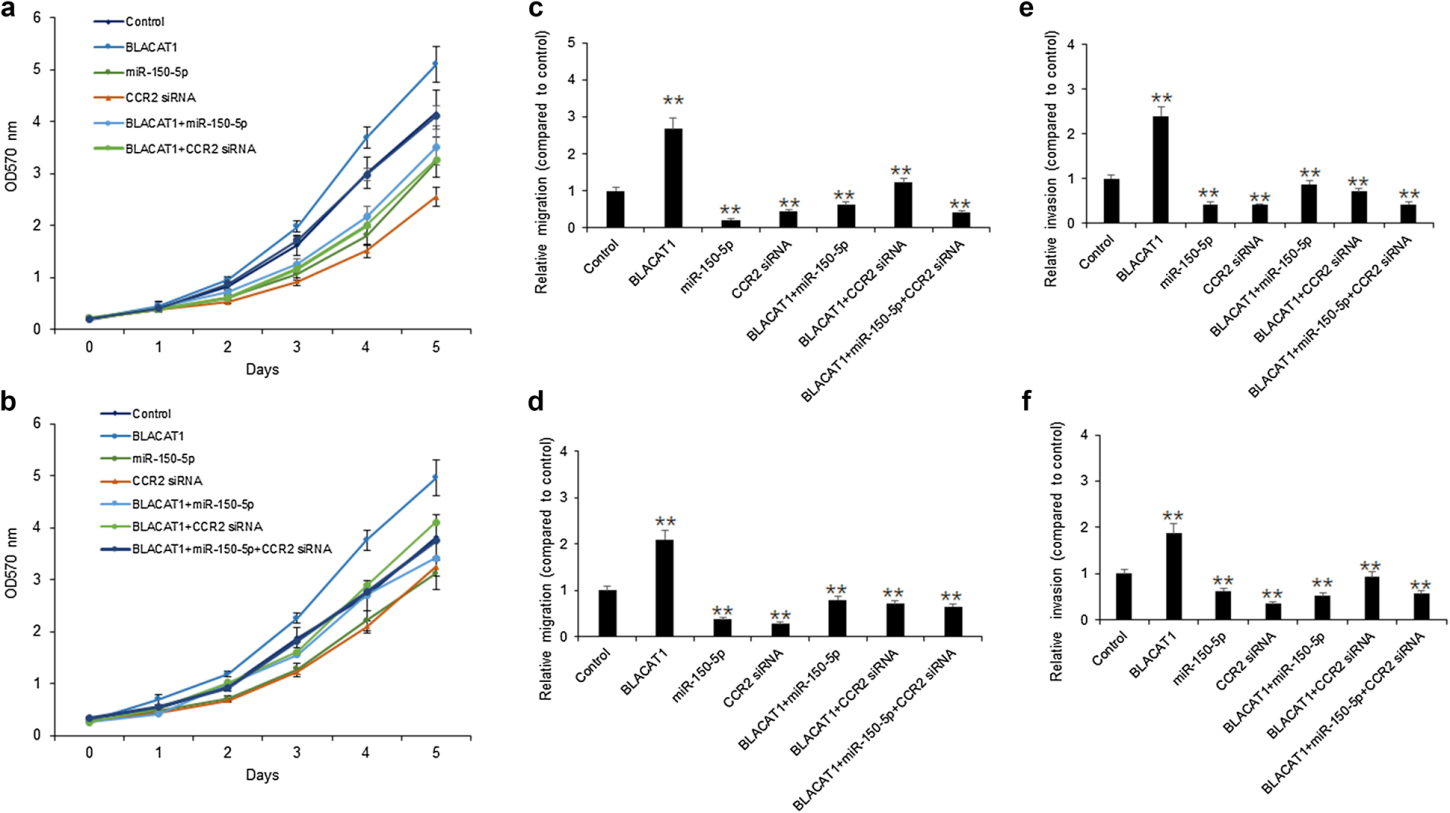
BLACAT1 promoted breast cancer cell proliferation and metastasis by miR-150-5p/CCR2. **a**, **b** SKBR3 and MDA-MB-231 cells were transfected with BLACAT, miR-150-5p or CCR2 siRNAs, and the MTT assay was used to analyze cell survival ability. **c**, **d** SKBR3 and MDA-MB-231 cells were transfected with BLACAT, miR-150-5p or CCR2 siRNAs, and the transwell system was used to analyze cell invasion ability

To know whether BLACAT influences breast cancer cell functions via miR-150-5p/CCR2, MTT assay was used to assess cell survival of SKBR3 and MDA-MB-231 cells. SKBR3 and MDA-MB-231 cells were transfected with BLACAT, miR-150-5p or CCR2 siRNAs, and the MTT assay result showed that BLACAT promoted cell survival ability in the cells with miR-150-5p or CCR2 siRNA transfection (Fig. 5a, b). The data from invasion assay showed that BLACAT promoted cell invasion ability in the cells with miR-150-5p or CCR2 siRNA transfection (Fig. 5c, d). The data indicated that BLACAT1 promoted breast cancer cell proliferation and metastasis by miR-150-5p/CCR2.

### The clinic relationship between BLACAT1, miR-150-5p and CCR2 in breast cancer tissues

**Fig. 6:**
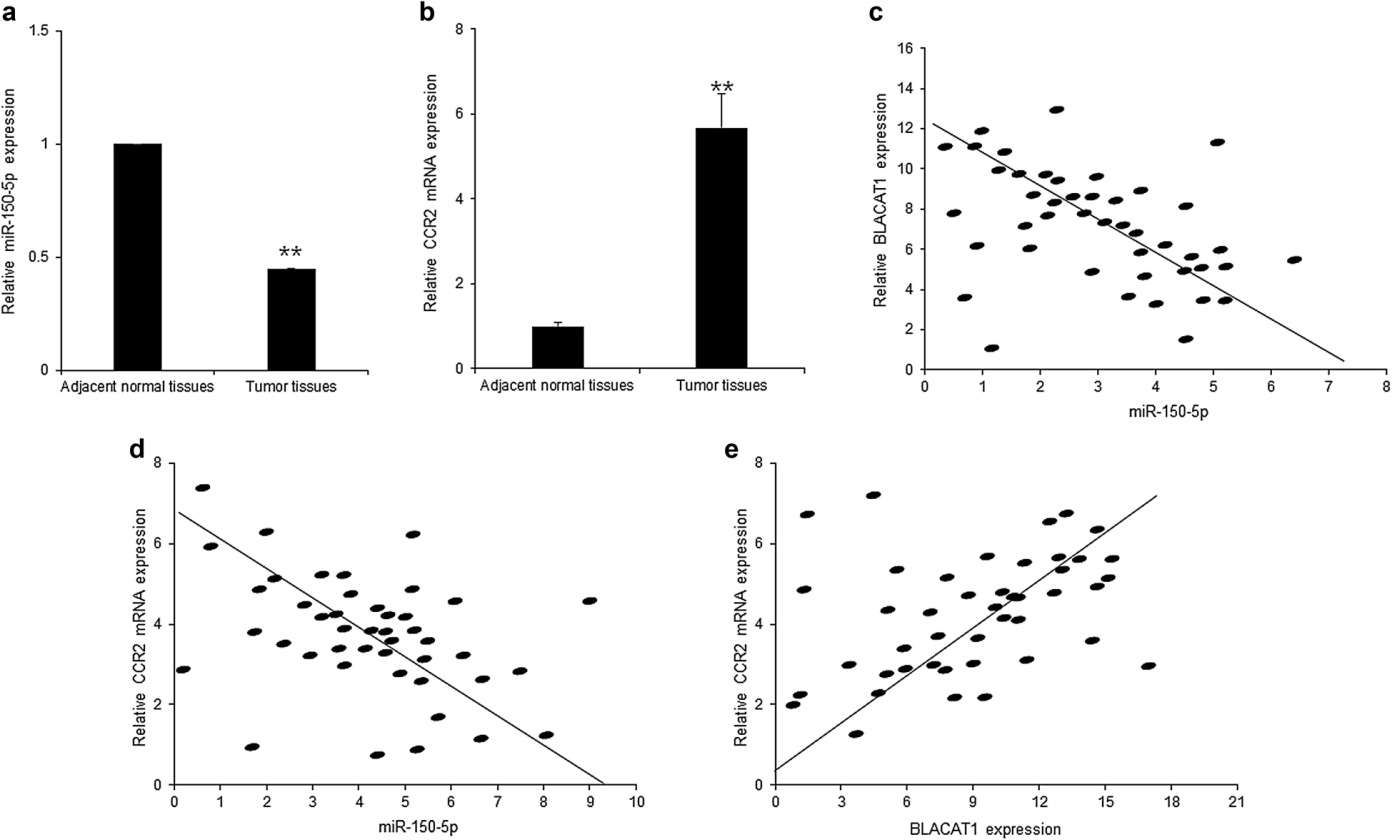
The clinic relationship between BLACAT1, miR-150-5p and CCR2 in breast cancer tissues. **a** MiR-150-5p was downregulated in 72 primary breast cancer tissues in contrast to paired nontumor tissues. **b** CCR2 expression was higher in 72 primary breast cancer tissues in contrast to paired nontumor tissues. **c** The relationship between BLACAT1 and miR-150-5p in breast cancer cell lines (r = − 0.458). **d** The relationship between BLACAT1 and CCR2 mRNA in breast cancer cell lines (r = − 0.517). **e** The relationship between miR-150-5p and CCR2 mRNA in breast cancer cell lines (r = − 0.379)

The above data showed that BLACAT1 had a higher level in breast cancer tissues and cells and was associated with metastasis. Here, the relationship between BLACAT1, miR-150-5p and CCR2 expression in breast cancer was analyzed. MiR-150-5p and CCR2 mRNA expression was shown in Fig. 6a, b. As the same result from breast cancer cells, miR-150-5p was lower in breast cancer tissues than the adjacent normal tissues (Fig. 6a). CCR2 mRNA was higher in breast cancer tissues than the adjacent normal tissues (Fig. 6b). Further data analysis showed that BLACAT1 was negatively related to miR-150-5p expression (Fig. 6c) and positively related to CCR2 mRNA levels (Fig. 6d) in breast cancer tissues. miR-150-5p was negatively related CCR2 mRNA in in breast cancer tissues (Fig. 6e).

## Discussion

In the past, extensive efforts have contributed to investigate the molecular and cellular mechanisms of breast cancer. There are great achievements in exploring the potential therapeutic targets, however, there is long way to find the effective target. lncRNAs may be used as potential prognostic markers and therapeutic targets of breast cancer. The present data demonstrated that lncRNA-BLACAT1 was upregulated in breast cancer, which promoted breast cancer cell proliferation and metastasis via down-regulating miR-150-5p by targeting CCR2 expression.

Long-coding RNAs are new regulators in gene expression and thus biological and pathophysiological processes. The bioinformatics tools and high**-**throughput sequencing are developed rapidly and many potential lncRNAs have been identified. But the mechanisms are unclear. Emerging reports have indicated the important function of lncRNAs in the development of breast cancer [15]. For example, long non-coding RNA SNHG14 induced breast cancer cell trastuzumab resistance by regulating PABPC1 expression through H3K27 acetylation [16]. LncRNA MIR100HG promoted cell proliferation in triple-negative breast cancer through triplex formation with p27 loci [17]. Exosome-mediated transfer of lncRNA–SNHG14 promoted trastuzumab chemoresistance in breast cancer [18]. LncRNA ITGB2-AS1 promoted the migration and invasion of breast cancer cells through up-regulating ITGB2 [19]. In the study, we found that the expression of BLACAT1 in breast cancer tissue/cells was increased, and its expression levels were related to the breast cancer metastasis, staging and survival. However, if the BLACAT1 would be a prognostic maker, BLACAT1 levels in circulation will be detected in the breast cancer patients.

The most canonical theory for lncRNAs is the miRNAs “sponge”, performing as miRNAs absorbers to specifically attenuate the miRNAs abundance. For examples, LOC101930370/miR-1471 axis regulated breast cancer progression via the hedgehog signaling pathway [20]; LncRNA GAS5-MiR-23a-ATG3 axis regulated autophagy in breast cancer patients [21]; Linc00518 contributed to multidrug resistance through regulating the MiR-199a/MRP1 axis in breast cancer [22]; Downregulation of lncRNA GAS5 conferred tamoxifen resistance by activating miR-222 in breast cancer [23]; LncRNA ARNILA induced negatively by an androgen receptor bond to miR-204 and promoted the invasion and metastasis of triple-negative breast cancer [24]. Our team investigated the potential regulatory pathway of BLACAT1 in breast cancer cells. Results indicate that miR**-**150-5p binds with the 3′**-**UTR of CCR2, which was confirmed using luciferase reporter assay. Moreover, miR**-**150-5p also targets with 3′**-**UTR of CCR2 mRNA. Our results reveal that BLACAT1 promoted CCR2 expression on post-transcriptional levels through sponging miR**-**150-5p in breast cancer.

In conclusion, above evidence reveals the vital role of lncRNA BLACAT1 in the growth and metastasis of breast cancer. BLACAT1 promotes human breast cancer tumorigenesis by targeting miR**-**150-5p/CCR2 axis, suggesting the novel molecular mechanism of breast cancer.
